# Metformin prevents stroke damage in non-diabetic female mice with chronic kidney disease

**DOI:** 10.1038/s41598-021-86905-9

**Published:** 2021-04-02

**Authors:** Maria Grissi, Cédric Boudot, Maryam Assem, Alexandre Candellier, Mathilde Lando, Sabrina Poirot-Leclercq, Agnès Boullier, Youssef Bennis, Gaëlle Lenglet, Carine Avondo, Jean-Daniel Lalau, Gabriel Choukroun, Ziad A. Massy, Saïd Kamel, Jean-Marc Chillon, Lucie Hénaut

**Affiliations:** 1 UR UPJV 7517, MP3CV, CURS, Université de Picardie Jules Verne, Avenue René Laennec, 80054 Amiens, France; 2 Faculty of Medicine, University of Picardie Jules Verne, 80000 Amiens, France; 3 Division of Nephrology, Amiens University Hospital, 80054 Amiens, France; 4 Department of Biochemistry, Amiens University Hospital, 80054 Amiens, France; 5 Department of Clinical Pharmacology, Amiens University Hospital, 80054 Amiens, France; 6 Department of Endocrinology-Diabetology-Nutrition, Amiens University Hospital, 80054 Amiens, France; 7 UMR_I 01, PériTox, CURS, 80054 Amiens, France; 8 Department of Nephrology, Ambroise Paré University Hospital, APHP, 92104 Boulogne-Billancourt, France; 9 Inserm U1018-Team 5, CESP, UVSQ, University Paris Saclay, 94807 Villejuif, France; 10 University Versailles-Saint Quentin, University Paris-Saclay, 91190 Villejuif, France; 11 Faculty of Pharmacy, University of Picardie Jules Verne, 80000 Amiens, France; 12 Direction of Clinical Research, Amiens University Hospital, 80054 Amiens, France

**Keywords:** Diseases, Nephrology, Neurology

## Abstract

Chronic kidney disease (CKD) worsens ischemic stroke severity in both patients and animals. In mice, these poorer functional outcomes are associated with decreased brain activity of AMP-activated protein kinase (AMPK), a molecule that recently emerged as a potential therapeutic target for ischemic stroke. The antidiabetic drug metformin, a well-known activator of AMPK, has improved stroke outcomes in diabetic patients with normal renal function. We investigated whether chronic metformin pre-conditioning can rescue AMPK activity and prevent stroke damage in non-diabetic mice with CKD. Eight-week-old female C57BL/6J mice were assigned to CKD or SHAM groups. CKD was induced through right kidney cortical electrocautery, followed by left total nephrectomy. Mice were then allocated to receive metformin (200 mg/kg/day) or vehicle for 5 weeks until stroke induction by transient middle cerebral artery occlusion (tMCAO). The infarct volumes were lower in CKD mice exposed to metformin than in vehicle-treated CKD mice 24 h after tMCAO. Metformin pre-conditioning of CKD mice improved their neurological score, grip strength, and prehensile abilities. It also enhanced AMPK activation, reduced apoptosis, increased neuron survival and decreased microglia/macrophage M_1_ signature gene expression as well as CKD-induced activation of the canonical NF-κB pathway in the ischemic lesions of CKD mice.

## Introduction

Stroke is the third leading cause of death from cardiovascular disease in patients with chronic kidney disease (CKD)^1^. Around 75% of all strokes experienced by dialysis patients are ischemic strokes^1–3^. The high frequency of stroke observed in this population may be explained by the high prevalence of both traditional cardiovascular risk factors (such as dyslipidemia, diabetes and hypertension) and non-traditional risk factors related to impaired kidney function (such as disorders of bone and mineral metabolism, oxidative stress, and the accumulation of uremic toxins). Therefore, a linear and additive rise in the risk of stroke is observed in response to declining glomerular filtration rate and increasing proteinuria^3,4^. Advanced CKD has been associated with a higher risk of neurological deterioration, in-hospital mortality, and poor functional outcomes following acute ischemic stroke^5^. To date, the processes by which CKD worsens the severity of ischemic stroke are not fully understood and therapeutic strategies aiming to prevent stroke severity in these patients are missing^6^.

Metformin, a first-line drug for glycemic control in patients with type-2 diabetes mellitus (T2DM)^7^, has been shown to markedly reduce the risk of ischemic stroke in diabetic patients with normal renal function^8,9^. In these patients, glycemic control with metformin prior to the development of stroke is associated with reduced neurological severity and improved acute-phase outcomes^9^. Experimental data obtained from non-diabetic animals with normal renal function demonstrated that these beneficial effects mainly depend on the activation of adenosine-monophosphate-activated protein kinase (AMPK)^10,11^, a molecule that recently emerged as a potential therapeutic target for ischemic stroke^12^. Among the main mechanisms involved, AMPK activation has been reported to inhibit neuroinflammation^13–15^, decrease oxidative stress^14,16,17^, promote autophagy^18^, reduce blood–brain barrier disruption^19^, restrain glutamate release and excitotoxicity^20–22^, improve mitochondrial dysfunction^23^, and inhibit apoptosis in ischemic stroke^12,18^.

Our group recently observed that ischemic lesions from CKD mice, which are wider and more inflammatory, also show lower AMPK activity than that of mice with normal renal function^24^, suggesting the existence of a causative link between AMPK activity and stroke severity in CKD. To date, preclinical studies evaluating the effect of metformin on stroke recovery in the CKD setting have never been undertaken due to the fear of lactic acidosis^25^. In this context, the recent observations that metformin can be safely used in patients with advanced CKD without increasing the risk of lactic acidosis, provided that the dose of metformin is adapted to kidney function, opened up new perspectives in the prevention of stroke severity in this population^26,27^. The present work aimed to evaluate whether metformin can be used in non-diabetic CKD mice to rescue AMPK activity and prevent stroke severity.

## Results

### Body weight, serum biochemistry and hematology

Table 1 presents data on bodyweight, as well as serum biochemistry and hematology parameters. Serum urea, phosphorus, and calcium concentrations were significantly higher in CKD-veh than SHAM-veh mice. Hematology parameters, in particular the hemoglobin concentration, red blood cell count and hematocrit, were significantly lower in CKD-veh than SHAM-veh mice. Before stroke induction, the CKD-veh mice had a lower mean bodyweight than the SHAM-veh mice. Post-stroke bodyweight loss was not significantly different between the CKD-veh and SHAM-veh mice. Plasma metformin concentrations were significantly higher in CKD-Met than in SHAM-Met mice (0.53 ± 0.21 mg/L vs. 0.27 ± 0.14 mg/L, p < 0.01). This difference was not associated with an increase in lactate concentration (Table 1). There were no differences in biochemical/hematological parameters or mean bodyweight between vehicle- and metformin-treated mice.

**Table 1:** Routine serum biochemistry and hematology parameters.

	Reference values for mice	SHAM-veh	SHAM-Met	CKD-veh	CKD-Met
**Serum biochemistry**					
Urea (mmol/L)	2.85–11.78	9.017 ± 2.210	8.913 ± 2.104	32.210 ± 10.560*	27.380 ± 7.310^$^
BUN (mg/dL)	10–33	26.690 ± 8.900	24.970 ± 5.893	90.23 ± 29.570*	76.690 ± 20.480^$^
Phosphorous (mmol/L)	1.98–3.01	2.984 ± 0.518	3.127 ± 0.570	3.772 ± 0.817*	3.303 ± 0.589
Calcium (mmol/L)	1.77–2.52	2.416 ± 0.123	2.387 ± 0.099	2.713 ± 0.124*	2.694 ± 0.119^$^
Metformin (mg/L)		–	0.27 ± 0.14	–	0.53 ± 0.21^$$^
Lactate (mmol/L)		2.760 ± 1.860	2.520 ± 1.384	3.8 ± 1.524	3.470 ± 1.679
**Hematology**					
Hemoglobin (g/dL)	10–20	12.360 ± 1.230	13.210 ± 0.644	9.369 ± 1.926*	10.530 ± 0.875^$^
Red blood cells (× 10^12^/L)	7–11	6.723 ± 0.568	7.1 ± 0.433	5.222 ± 0.954*	5.873 ± 0.511^$^
Hematocrit (%)	35–40	39.95 ± 3.497	42.27 ± 2.795	29.73 ± 5.478*	33.43 ± 3.295^$^
**Body weight**					
Before tMCAO		21.02 ± 1.512	21.63 ± 1.333	19.37 ± 1.044*	19.25 ± 0.6308^$^
After tMCAO		18.88 ± 1.498	20.21 ± 1.402	17 ± 1.095*	17.17 ± 1.103^$^
% of weight loss		10.17 ± 3.101	6.624 ± 4.969	12.19 ± 4.015	10.26 ± 3.897

*BUN* blood urea nitrogen, *CKD* chronic kidney disease, *CKD-veh* CKD-vehicle, *CKD-met* CKD metformin, *SHAM-veh* SHAM-vehicle, *SHAM-met* SHAM Metformin. Data are expressed as the mean ± SD. *p < 0.05, CKD-veh vs. SHAM-veh mice. ^$^p < 0.05, ^$$^p < 0.01, CKD-met vs. SHAM-met mice.

### Metformin pre-conditioning reduces infarct volume and neurological deficits after tMCAO in CKD mice

**Figure 1:**
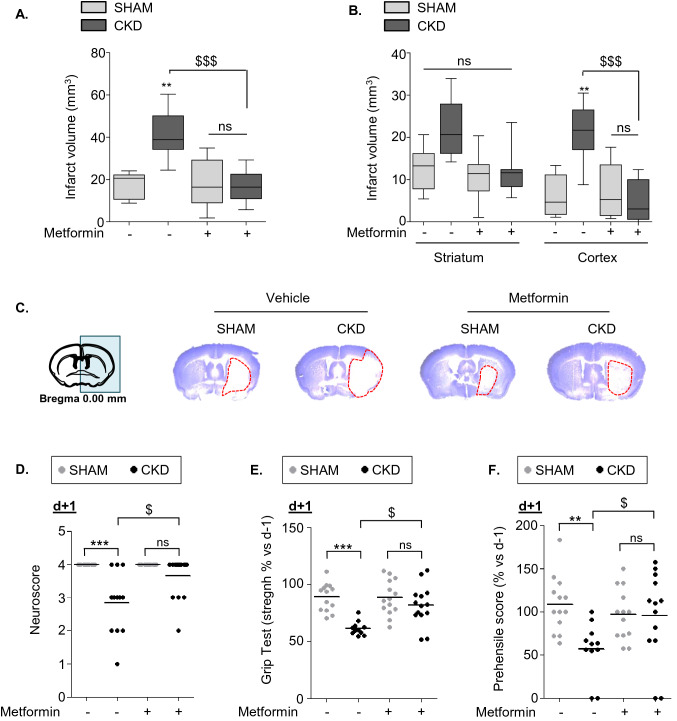
Metformin pre-conditioning reduces infarct volume and neurological deficits after tMCAO in CKD mice. (**A**–**C**) Analysis of total (**A**), as well as cortical and striatal (**B**), infarct volumes in SHAM and CKD animals exposed, or not, to metformin. Representative images of cresyl-violet staining are presented in (**C**). Results are expressed as the median, interquartile, and min–max. n = 7 SHAM-veh, n = 14 CKD-veh, n = 8 SHAM-met, n = 12 CKD-met. Statistical analysis was performed using a non-parametric Kruskall–Wallis test followed by Dunn’s multiple comparison post-hoc test. **p < 0.01, CKD-vehicle vs. SHAM-vehicle mice (non-parametric Mann–Whitney U test). ^$$$^p < 0.001, CKD-metformin vs. CKD-vehicle mice (non-parametric Mann–Whitney *U* test). (**D**–**F**) Neurobehavioral evaluation performed 24 h after tMCAO. n = 13 SHAM-veh, n = 11–12 CKD-veh, n = 14 SHAM-met, n = 14 CKD-met. Statistical analysis was performed using a non-parametric Kruskall–Wallis test followed by Dunn’s multiple comparison post-hoc test. **p < 0.01 and ***p < 0.001, CKD-vehicle vs. SHAM-vehicle mice. ^$^p < 0.05, CKD-metformin vs. CKD-vehicle mice.

The total volume of ischemic damage was significantly higher in CKD-veh than SHAM-veh mice (CKD-veh: 41.73 ± 10.81 mm^3^ vs. SHAM-veh: 17.59 ± 5.96 mm^3^, p < 0.01) (Fig. 1A). Metformin pre-conditioning significantly reduced the total volume of ischemic damage of CKD mice relative to that of vehicle-treated animals (CKD-met: 16.85 ± 7.06 mm^3^ vs. CKD-veh: 41.73 ± 10.81 mm^3^, p < 0.001) (Fig. 1A,C). However, metformin treatment did not affect infarct volumes in SHAM-operated mice (SHAM-met: 18.28 ± 11.33 vs. SHAM-veh: 17.59 ± 5.96, ns). The infarcted area in SHAM-veh mice was mainly located within the striatum (ischemic core), whereas the volume of cortical infarction (ischemic penumbra) was limited (Fig. 1B,C). CKD mice showed significantly higher cortical infarct volumes than SHAM-operated mice (CKD-veh: 21.16 ± 6.30 mm^3^ vs. SHAM-veh: 5.67 ± 4.77 mm^3^, p < 0.01). Pre-conditioning with metformin significantly reduced the cortical damage in CKD mice (CKD-met: 4.93 ± 4.66 mm^3^ vs. CKD-veh: 21.16 ± 6.30 mm^3^, p < 0.001) (Fig. 1B,C). Pre-conditioning with metformin did not influence striatal infarct volumes in any of the groups.

Twenty-four hours after stroke induction, the neurological score, grip strength, and prehensile abilities of the CKD-veh mice were significantly lower than those of the SHAM-veh mice (p < 0.01) (Fig. 1D–F). Metformin pre-conditioning of CKD mice significantly improved their neurological scores (CKD-met: 3.64 ± 0.63 vs. CKD-veh: 2.83 ± 0.93*,* p < 0.05), grip strength (CKD-met: 81.51 ± 17.45% vs. CKD-veh: 61.44 ± 6.03%*,* p < 0.05), and prehensile capacity (CKD-met: 94.88 ± 51.10% vs. CKD-veh: 55.06 ± 30.72%*,* p < 0.05) relative to pre-conditioning with the vehicle (Fig. 1D–F). Metformin pre-conditioning did not affect the functional damage of SHAM-operated mice.

### Metformin pre-conditioning reduces both apoptosis and neuronal loss in the cortex of CKD mice

**Figure 2:**
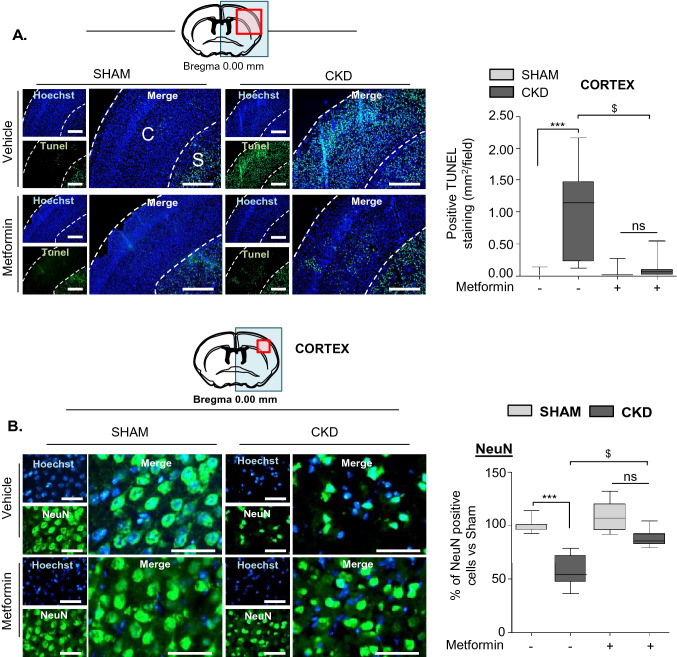
Metformin pre-conditioning reduces both apoptosis and neuronal loss in the cortex of CKD mice. (**A**) The left panels show representative images of the TUNEL immunostaining performed on SHAM and CKD mice exposed, or not, to metformin. Scale bars: 500 µm. Quantification of TUNEL immunostaining of the cortex is presented on the right side of the figure. Quantifications show the amount of TUNEL-positive surface per 40,000-µm^2^ field. *C* Cortex ; *S* Striatum. (**B**) Immunostaining analysis of NeuN expression in the cortex of SHAM and CKD mice exposed, or not, to metformin. Scale bars: 50 µm. The quantifications presented on the right side of the figure show the percentage of NeuN-positive cells per 10,000-µm^2^ field. Results are expressed as the median, interquartile, and min–max and show data from at least 8 animals per group. Statistical analysis was performed using a non-parametric Kruskall–Wallis test followed by Dunn’s multiple comparison post-hoc test. ***p < 0.001, CKD-vehicle vs. SHAM-vehicle mice. ^$^p < 0.05 CKD-metformin vs. CKD-vehicle mice.

We assessed apoptosis in the cortex of both SHAM and CKD animals by TUNEL staining (Fig. 2A). The cortex of SHAM animals contained very few TUNEL-positive nuclei, whereas the surface of the cortex of CKD animals containing apoptotic cells was 50-fold higher (CKD-veh: 0.973 ± 0.645 mm^2^/field vs. SHAM-veh: 0.017 ± 0.039 mm^2^/field, p < 0.001). Metformin pre-conditioning of CKD mice significantly reduced cortical apoptosis relative to treatment with the vehicle (CKD-met: 0.115 ± 0.159 vs. CKD-veh: 0.973 ± 0.645 mm^2^/field, p < 0.05). Cortical apoptosis was not significantly different between SHAM-met and CKD-met mice (SHAM-met: 0.043 ± 0.099 vs. CKD-met: 0.115 ± 0.159 mm^2^/field, ns). Neither CKD, nor metformin pre-conditioning modified TUNEL staining in the striatum (Supplementary Figure 2). The number of NeuN-positive cells in the cortex of CKD-veh mice was significantly lower than that in SHAM-veh mice (CKD-veh: 57.76 ± 13.71 vs. SHAM-veh: 100 ± 6.15% of NeuN-positive cells, p < 0.001) (Fig. 2B). The number of NeuN-positive cells in the cortex of CKD mice treated with metformin was significantly higher than that in vehicle-treated animals (CKD-met: 87.92 ± 7.51 vs. CKD-veh: 57.76 ± 13.71% of NeuN-positive cells, p < 0.001) (Fig. 2B). The number of NeuN-positive cells found in CKD-met and SHAM-met animals was not significantly different.

### Metformin pre-conditioning decreases microglia/macrophage recruitment and reduces the expression of M_1_ signature genes in the ischemic lesions of CKD mice

**Figure 3:**
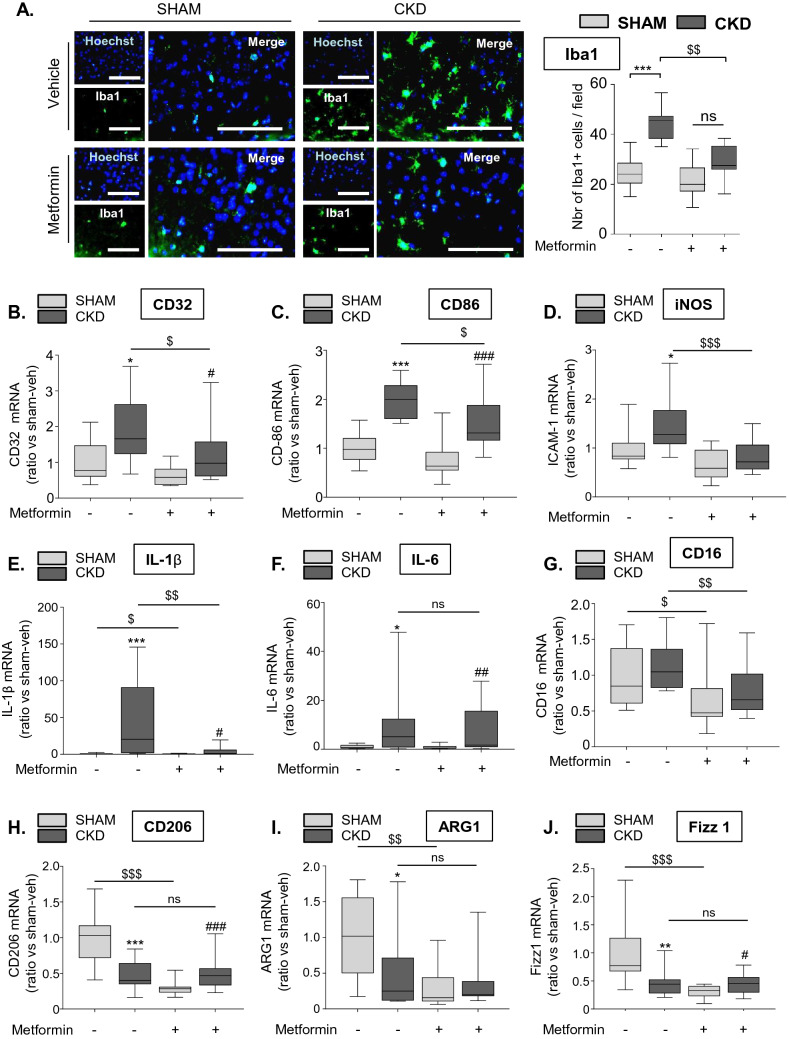
Metformin pre-conditioning decreases microglia/macrophage recruitment and reduces the expression of M_1_ signature genes in the ischemic lesions of CKD mice. (**A**) Immunostaining analysis of Iba-1 expression (a marker of activated microglia) in the cortex of SHAM and CKD mice treated, or not, with metformin. Scale bars: 100 µm. Quantifications show the number of Iba-1-positive cells per 10,000-µm^2^ field. Results are expressed as the median, interquartile, and min–max and show data from at least 8 animals per group. Statistical analysis was performed using a non-parametric Kruskall–Wallis test followed by Dunn’s multiple comparison post-hoc test. ***p < 0.001, CKD-vehicle vs. SHAM-vehicle mice. ^$$^p < 0.01, CKD-metformin vs. CKD-vehicle mice. (**B**–**G**) Real-time PCR analysis of the M_1_ markers CD32 (**B**), CD86 (**C**), iNOS (**D**), IL-1β (**E**), IL-6 (**F**) and CD16 (**G**). (**H**–**J**) Real-time PCR analysis of the M_2_ markers CD206 (**H**), ARG1 (**I**), and FIZZ1 (**J**). Results are expressed as the median, interquartile, and min–max and show data from at least 8 animals per group. Statistical analysis was performed using a non-parametric Kruskall–Wallis test followed by Dunn’s multiple comparison post-hoc test. *p < 0.05, **p < 0.01, ***p < 0.001, CKD-vehicle vs. SHAM-vehicle mice. ^#^p < 0.05, ^##^p < 0.01, ^###^p < 0.001, CKD-metformin vs. SHAM-metformin mice. ^$^p < 0.05, ^$$^p < 0.01, ^$$$^p < 0.001, CKD-metformin vs. CKD-vehicle mice.

We performed immunohistological analysis of ionized calcium-binding adapter molecule 1 (Iba1), a calcium-binding adapter protein that labels both monocytes and resting and activated microglia, to evaluate the recruitment of microglia/macrophages in ischemic lesions. We observed significantly more cortical Iba1-positive cells in the ischemic lesions of CKD-veh than those of SHAM-veh mice (CKD-veh: 44.30 ± 6.25 vs. SHAM-veh: 24.53 ± 6.47 Iba1-positive cells/field, p < 0.001) (Fig. 3A). Exposure to metformin significantly reduced the recruitment of Iba1-positive cells in CKD mice compared to exposure to the vehicle (CKD-met: 28.99 ± 6.57 vs. CKD-veh: 44.3 ± 6.25 Iba1-positive cells/field, p < 0.01). The recruitment of Iba1-positive cells was not significantly different between SHAM-met and CKD-met mice (SHAM-met: 21.21 ± 6.88 vs. CKD-met: 28.99 ± 6.57 Iba1-positive cells/field, ns).

The expression of the M_1_ signature genes CD32, CD86, iNOS, IL-1β, and IL-6 (Fig. 3B–F) was significantly higher in the ischemic lesions of CKD-veh mice than those of SHAM-veh mice 24 h after tMCAO. Metformin pre-conditioning of CKD animals significantly reduced CKD-induced mRNA expression of CD32 (p < 0.05), CD86 (p < 0.05), iNOS (p < 0.001), and IL-1β (p < 0.01) (Fig. 3B–E), as well as that of the M1 marker CD16 (p < 0.01) (Fig. 3G) relative to that of vehicle treated CKD mice. Pre-conditioning with metformin did not reduce CKD-induced IL-6 mRNA expression relative to that of vehicle treated mice (Fig. 3F). There was no difference in TNF-α mRNA expression between the ischemic hemispheres of CKD and SHAM-operated mice treated, or not, with metformin (Supplementary Figure 3). The M_2_ markers CD206, ARG1, and Fizz1 were significantly lower in the ischemic lesions of CKD-veh than SHAM-veh animals (Fig. 3H–J, p < 0.001, p < 0.05 and p < 0.01, respectively). Metformin treatment did not affect the reduction of M_2_ markers induced by CKD (Fig. 3H–J).

### Metformin pre-conditioning enhances adenosine monophosphate-activated protein kinase (AMPK) activation and reduces canonical NFκB activation in CKD mice

**Figure 4:**
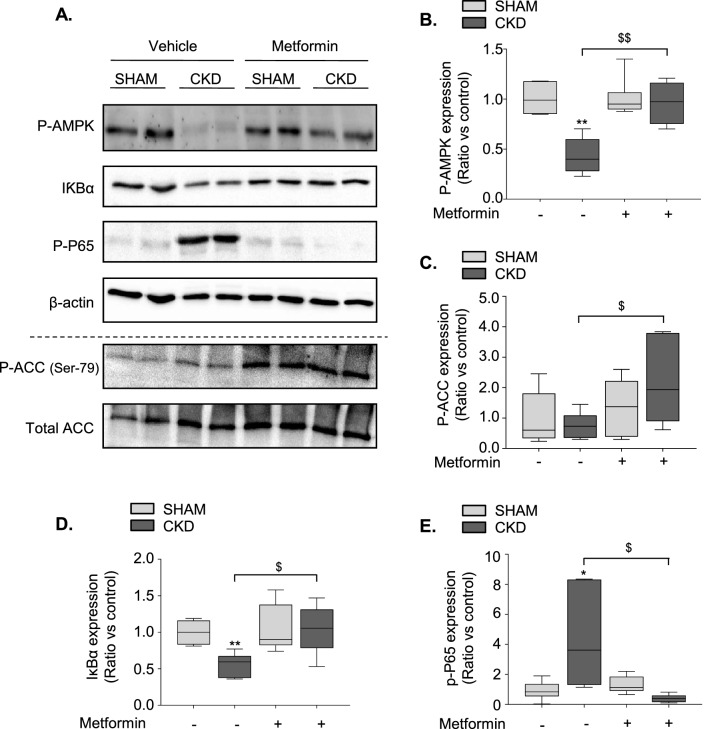
Metformin pre-conditioning enhances adenosine monophosphate-activated protein kinase (AMPK) activation and reduces canonical NFκB activation in CKD mice. (**A**) Representative images of phosho-AMPK, IΚBα, phospho-P65, phospho-ACC (Ser-79) and total-ACC western blots performed on SHAM and CKD mice exposed, or not, to metformin. (**B**) Quantitative data showing higher AMPK phosphorylation in ischemic hemispheres of CKD-metformin than CKD-vehicle treated mice. (**C**) Quantitative data showing higher ACC phosphorylation in ischemic hemispheres of CKD-metformin than CKD-vehicle treated mice. (**D**) Quantitative data showing higher IΚBα expression in ischemic hemispheres of CKD-metformin than CKD-vehicle treated mice. (**E**) Quantitative data showing lower P65 phosphorylation in ischemic hemispheres of CKD-metformin than CKD-vehicle treated mice. Results are expressed as the median, interquartile, and min–max and show data from at least 6 animals per group. Statistical analysis was performed using a non-parametric Mann–Whitney test. *p < 0.05, **p < 0.01, CKD-vehicle vs. SHAM-vehicle mice. ^$^p < 0.05, ^$$^p < 0.01, CKD-metformin vs. CKD-vehicle mice.

AMPK phosphorylation has been reported to impair M_1_ and favor M_2_ polarization of microglia/macrophages through the downregulation of NF-κB signaling^28–30^. Our group previously reported reduced AMPK activation in the ischemic brain of CKD animals^24^. We thus examined whether metformin pre-conditioning rescues AMPK phosphorylation in these animals. Western blot analysis showed significantly less AMPK phosphorylation in the ischemic brain of CKD-veh than SHAM-veh mice (p < 0.01) (Fig. 4A,B). Metformin pre-conditioning significantly increased the phosphorylation of both AMPK (p < 0.01) and its downstream target acetyl-coA carboxylase (ACC) (p < 0.05) in CKD mice relative to that of vehicle-treated mice (Fig. 4A–C). Degradation of IκBα and phosphorylation of P65 are hallmarks of NFκB pathway activation^31^. CKD-veh mice showed lower IκBα expression (p < 0.01) and higher P65 phosphorylation (p < 0.05) than SHAM-veh mice (Fig. 4A,D,E). Metformin pre-conditioning of CKD mice rescued IκBα expression and abolished the induction of P65 phosphorylation (Fig. 4A,D,E, p < 0.05). Metformin pre-conditioning did not affect the expression of Phospho-AMPK, Phospho-ACC, IΚBα, or Phospho-P65 in SHAM-operated mice

### Metformin pre-conditioning decreases astrogliosis in the ischemic penumbra of CKD mice

**Figure 5:**
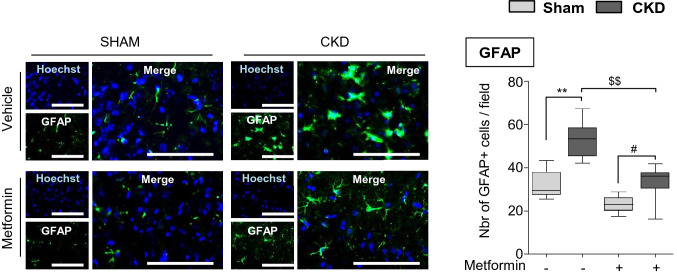
Metformin pre-conditioning decreases astrogliosis in the ischemic penumbra of CKD mice. Immunostaining analysis of GFAP expression (a marker of astrocytes) in the cortex of SHAM and CKD mice exposed, or not, to metformin. Scale bars: 100 µm. Quantifications show the number of GFAP positive cells per 10,000-µm^2^ field. Results are expressed as the median, interquartile, and min–max and show data from at least 8 animals per group. Statistical analysis was performed using a non-parametric Kruskall–Wallis test followed by Dunn’s multiple comparison post-hoc test. **p < 0.01, CKD-vehicle vs. SHAM-vehicle mice. ^#^p < 0.05, CKD-metformin vs. SHAM-metformin mice. ^$$^p < 0.01, CKD-metformin vs. CKD-vehicle mice.

Immunohistological analysis of glial fibrillary acidic proteins (GFAPs), markers of activated astrocytes, showed a significantly higher number of hypertrophic astrocytes in the ischemic cortex of CKD-veh than SHAM-veh mice (CKD-veh: 52.66 ± 7.80 vs. SHAM-veh: 32.02 ± 6.41 GFAP-positive cells/field, p < 0.01) (Fig. 5). Metformin pre-conditioning significantly reduced this effect (CKD-met 33.65 ± 6.43 vs. CKD-veh 52.66 ± 7.80 GFAP-positive cells/field, p < 0.01).

## Discussion

**Figure 6:**
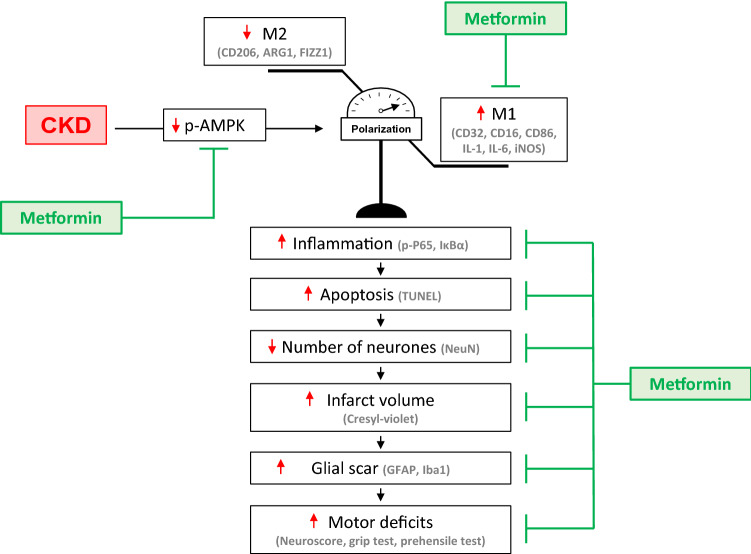
Hypothetical scheme of the mechanisms by which metformin prevent ischemic stroke damage in non-diabetic mice with CKD. In brain ischemic lesions of non-diabetic CKD mice, AMPK activity, which is known to block microglia/macrophages M1 polarization, is impaired. The recruited microglia/macrophages consequently display increased M1 and reduced M2 polarization. The subsequent inflammation may be responsible for the elevated apoptosis, increased infarct volumes and poorer functional outcomes. A 5-week course of pre-conditioning with metformin rescued the activation of AMPK in mice with CKD. This effect is associated with a significant decrease of macrophage/microglia transition towards the M_1_ phenotype, as shown by the decreased expression of the M_1_ markers CD16, CD32, and CD86. Metformin-induced AMPK activation blocks NFκB activation and the subsequent release of pro-inflammatory cytokines such as IL-1β. This decrease in inflammation may be responsible for less brain-cell apoptosis, thus reducing the cortical infarct volume and subsequent neurobehavioral disorders.

The present study demonstrates, for the first time to our knowledge, that metformin prevents stroke severity in non-diabetic mice with CKD. We previously postulated that the decrease of AMPK activity observed in non-diabetic CKD mice may be responsible for stroke severity^24^. Here, we demonstrate that chronic pre-conditioning with metformin efficiently rescues AMPK activation in the ischemic brain of CKD mice. We further provide evidence suggesting that metformin’s beneficial effect on CKD-enhanced stroke damage is linked to the reduction of CKD-induced microglia/macrophage M_1_ polarization and the subsequent reduction of inflammation. These data are in accordance with those of recent studies showing that metformin can rapidly penetrate the blood–brain barrier and differentially accumulate in the brain, where it can help to attenuate inflammation^13,32,33^. The main results are summarized in Fig. 6.

In the present study, plasma urea levels of vehicle-treated mice (SHAM and CKD) negatively correlated with both AMPK activation and IΚBα expression (Supplementary Figure 4A,B) and positively correlated with P65 phosphorylation (Supplementary Figure 4C) and the expression of the M_1_ markers CD86, iNOS, and IL-1β (Supplementary Figure 4D–F). Overall, these data suggest that the higher the uremia, the lower the AMPK activity and the higher the inflammation at the lesion site. These data are in accordance with a previous report from Li et al*.* who showed that macrophages isolated from CKD rats display lower AMPK activity together with enhanced M_1_ polarization^34^. In their work, exposure of peritoneal and bone marrow derived macrophages from normal rats to uremic sera led to similar results, suggesting that CKD disturbs macrophages polarization through inhibition of the AMPK. To date, the reason why AMPK activity is impaired in CKD remains unknown.

We show that chronic metformin pre-conditioning rescued AMPK activation in CKD mice, an effect that was associated with a reduction of CKD-induced NF-κB pathway and a subsequent decrease in the level of the macrophage/microglia M_1_ markers CD32, CD16, CD86, iNOS, and IL-1β. These data are in accordance with those of recent studies showing that AMPK activation impairs macrophage/microglia polarization towards the M_1_ phenotype through the downregulation of NF-κB signaling^28–30,35–37^. They suggest that local reactivation of AMPK by metformin can rescue CKD-induced inflammation and subsequent brain damage by blocking macrophage/microglia M1 polarization at the lesion site. However, since the present study remains mainly observational, further investigations will be needed to confirm the existence of a causative link between metformin-induced activation of AMPK and metformin’s efficiency to reduce neuro-inflammation and stroke severity in CKD. Given that metformin can activate NRF2, which in turn blocks the activation of the NF-κB pathway^38^, the possibility that metformin attenuated both neuro-inflammation and stroke severity in an AMPK-independent manner cannot be excluded.

In a recent study, Jin et al*.* showed that metformin treatment (50 mg/kg/day given post-stroke for 30 days) skewed the microglia/macrophages toward a M_2_ phenotype following MCAO in CD1 mice, thereby promoting functional recovery^13^. They subsequently confirmed M_2_ polarization of BV2 microglial cells in vitro following metformin-induced AMPK activation. In our model, the beneficial effects of metformin preconditioning may only rely on the blockade of CKD-induced M_1_ polarization since the activation of AMPK did not rescue the CKD-induced decrease of microglia/macrophage M_2_ polarization.

In the present study, we could not discriminate between microglia and macrophage polarization, as all attempts to identify monocyte/macrophage infiltration and polarization by immunohistochemistry failed. However, given that uremia induces disruption of the blood–brain barrier, as well as the inflammatory cascade in cerebral tissues in other models^39^, it is very likely that we observed the polarization of a mixed population of microglia and freshly infiltrated monocytes/macrophages. Indeed, the levels of MCP1 (involved in monocytes chemotaxis), ICAM-1 and VCAM-1 (involved in monocytes adhesion and rolling) were higher in the ischemic hemispheres of CKD than SHAM animals (Supplementary Figure 5A–C). Interestingly, pre-exposure of CKD mice to metformin significantly lowered the expression of ICAM-1 (p < 0.01) and VCAM-1 (p < 0.01) relative to that of vehicle-treated CKD mice (Supplementary Figure 5A,B). This reduction of ICAM-1 and VCAM-1 levels may be responsible for the decreased recruitment of Iba-1-positive cells to the ischemic lesions of CKD mice exposed to metformin. This hypothesis is supported by the recent discovery that metformin (200 mg/kg) given post-stroke for 14 days can attenuate blood brain barrier disruption after tMCAO in male CD1 mice through the AMPK-dependent downregulation of ICAM-1^19^. The astrogliosis that takes place after a stroke is thought to worsen the ischemic damage because reactive astrocytes secrete growth inhibitory molecules, reactive oxygen species (ROS), pro-inflammatory cytokines, and MMPs^40,41^. In our model, metformin treatment blocked the reactive astrogliosis induced by CKD in the mouse cortex. These data are in accordance with those of a previous report by Zhu et al*.*, who showed that microgliosis and astrocytosis induced by permanent MCAO were improved by chronic metformin pre-conditioning (50 mg/kg/day for 3 weeks) in Sprague–Dawley rats^42^.

In a recent study, Jiang et al*.* showed that 24-h pre-conditioning with metformin activated AMPK-dependent autophagy in the brain, thereby conferring neuroprotection against focal cerebral ischemia in a rat model of permanent MCAO^18^. Indeed, pre-activation of autophagy in the brain was found to markedly enhance ischemic tolerance, as it facilitated cellular energy production, limited endoplasmic reticulum stress, and prevented neuronal apoptosis during subsequent ischemic exposure^43,44^. In our model, 5-week pre-conditioning with metformin induced autophagy in SHAM-operated mice, as shown by increased expression of LC3A/B-I and LC3A/B-II (Supplementary Figure 6A,B). However, metformin failed to improve autophagy in CKD mice, suggesting that the increased cell viability observed in response to metformin treatment in these mice does not depend on pre-activation of autophagy in the brain.

Neurogenesis, which allows the replacement of damaged neurons, favors stroke recovery^45^. Wang et al*.* reported that metformin can promote neurogenesis and enhance spatial memory formation in normal adult mice^46^. These data were later confirmed by Liu et al*.*, who showed that metformin (200 mg/kg), given daily after tMCAO, promoted neurogenesis and attenuated ischemia-induced brain injury in CD1 mice^47^. More recently, Yuan et al*.* showed reduced neuronal damage and increased neuroblast proliferation and differentiation in response to metformin in a cerebral ischemia/reperfusion rat model^48^. In our study, pre-conditioning with metformin did not rescue the impaired neurogenesis observed in ischemic lesions of CKD animals (Supplementary Figure 7). Further studies will be needed to determine whether the decline in AMPK activity observed in CKD mice directly influences neurogenesis.

The present results should be considered in the light of the metformin dose used, and the balance between the drug’s beneficial and adverse effects in the context of CKD. Although the mean metformin concentrations were higher in the serum of CKD-Met group compared to SHAM-Met mice (0.53 ± 0.21 mg/L vs. 0.27 ± 0.14 mg/L, p < 0.01), this difference was minimal and negligible with respect to the concentrations observed in clinical practice in metformin-induced lactic acidosis, where levels are in the order of dozens of milligrams per litre^49^. Moreover, there was no observed difference in lactate levels between both groups. Lastly, and importantly, rather than being “toxic” for the kidneys, a large bulk of clinical and experimental data strongly support the nephroprotective effect of metformin^50,51^ and several large observational studies have even shown a lower mortality rate in CKD patients treated with metformin^27,52^.

Metformin has been shown to have beneficial effects on the kidney in various clinical trials and experimental studies^51^. Metformin-induced preservation of renal function in adenine rats preserved mineral homeostasis in the circulation, which in turn prevented the development of vascular calcification and high bone turnover disease^53^. In accordance with these data, the induction of AMPK activity was shown to correct metabolic inefficiency of the kidney, improve kidney function, and reduce kidney fibrosis and structural alterations in 5/6 nephrectomized rats^54^. In this context, clinical trials repurposing metformin as a therapeutic strategy to prevent the progression of CKD in non-diabetic patients, are underway (NCT03831464, http://www.clinicaltrials.gov). Our data go beyond the kidney protection and shows a possible direct effect on stroke size and neurological consequences in non-diabetic CKD mice. In our model, metformin pre-conditioning did not affect renal function, as shown by the lack of modulation of serum urea, calcium, and phosphorus levels. The hematocrit and hemoglobin levels did not change in response to metformin treatment, showing that the kidneys were not able to preserve normal erythropoiesis. Therefore, the neuroprotective effects observed in our model cannot be attributed to nephroprotection but may be rather linked to the direct activation of AMPK in the brain. Further studies will be needed to confirm the exact cellular target of metformin, since all the western blots and PCRs presented in this study were performed on whole cell lysates. Whether metformin is able to mitigate the consequences of stroke occurring in non-diabetic CKD patients’ needs to be evaluated.

In this study, experiments were performed only on female mice. Therefore, the possibility that the estrous cycle and/or gonadal hormones may have influenced stroke recovery cannot be ruled out. Since AMPK is an energy sensor, evaluating the modulations of brain metabolism (e.g., glycolysis, glucose uptake, lipolysis, etc.) in response to metformin would have strengthened the data obtained in CKD mice. This can be considered as one of the limitations of the study.

High blood pressure is frequently associated with impaired kidney function and may influence ischemic stroke severity. In our model, the induction of CKD is not associated with hypertension^55–57^. Therefore, the impact of uremia on stroke recovery cannot be attributed to high blood pressure in this study.

## Conclusion

We show that metformin, given daily for 5 weeks prior to stroke induction, reduces brain inflammation, cortical infarct volume and neurobehavioral disorders in non-diabetic CKD mice. The present study suggests that metformin may have a practical clinical application for preventing stroke damage in CKD patients. Clinical studies are needed to explore whether metformin is able to mitigate the consequences of stroke occurring in non-diabetic CKD patients.

## Methods

### Animals

All experiments were performed on female C57BL/6J mice (Charles River Laboratories, Lyon, France). Animals were housed as previously described^24^. This work has been carried out in accordance with the UE Directive 2010/63/EU. The study was carried out in compliance with the ARRIVE guidelines. The protocol was approved by an institutional animal care committee (*Comité Régional d’Ethique en Matière d’Expérimentation Animale de Picardie*, Amiens, France) and the French Ministry of Education and Research (Protocol ID : APAFIS#7596). A schematic summary of the experimental protocol is presented in Supplementary Figure 1.

### Induction of chronic kidney disease

Animals were randomly assigned to CKD or SHAM groups at 8 weeks of age. CKD was induced as previously described^58^. Briefly, we applied cortical electrocautery to the right kidney through a 2-cm flank incision and then performed left total nephrectomy through a similar incision 2 weeks later^24^. Control animals underwent SHAM operations.

### Metformin treatment

One week after the CKD and SHAM surgeries, mice were randomly divided to receive, or not, metformin (200 mg/kg/day; Merck) dissolved in the drinking water, as described previously^59^. Experiments were therefore performed on four groups of mice: Sham-vehicle (Sham-veh), Sham-metformin (Sham-met), CKD-vehicle (CHD-veh), and CKD-metformin (CKD-met). The treatment was given daily for 5 weeks until stroke induction by transient middle cerebral artery occlusion (tMCAO).

### Transient middle cerebral artery occlusion (tMCAO)

Middle cerebral artery (MCA) occlusion was induced in all groups by the intraluminal filament method 5 weeks after the last SHAM or CKD surgery (i.e. in 15-week-old mice), as previously described^24^ (for details, see Supplementary Methods).

### Neurological evaluation

Neurological evaluations (neuroscore, prehensile test, and grip test) were performed daily, three days before (d-3, d-2, and d-1) and 24 h after stroke induction, as previously described^24^ (for details, see Supplementary Methods). No neurological evaluation was performed on the day of stroke induction (day 0). Only the scores obtained 24 h before and 24 h after stroke are presented here. The design of the study did not allow a blinded evaluation of the neurological impairments.

### Hematology and serum biochemistry

Blood samples were collected 24 h after MCAO and used for both hematology and serum biochemistry as previously described by our group^24^. Evaluation of the hematological parameters was performed on a Genius KT 6200 VET system (Shenzhen Genius Electronics Co., LTD) following the manufacturer’s instructions. The measurement of serum urea, phosphorus, calcium, and lactate levels was performed on a RX Daytona + system (Randox Laboratories). Metformin level in mice serum samples was determined using a validated method associating high performance liquid chromatography and photodiode array detection, as previously described^26^.

### Brain preparation for histology

Twenty-four hours after MCAO, mice were deeply anaesthetized by intraperitoneal administration of ketamine (80 mg/kg) and xylazine (8 m/kg). Euthanasia was performed by total exsanguination. Mice were then transcardially perfused with cold phosphate-buffered saline (PBS), followed by 4% paraformaldehyde (PFA). Brains were removed and post-fixed for 24 h in 4% PFA at 4 °C. Fixed brains were then dehydrated by incubation in PBS containing 20% sucrose for 24 h at 4 °C, followed by incubation in PBS containing 30% sucrose for 24 h at 4 °C. After freezing in isopentane, the brain tissue was coronally sectioned on a cryostat at 12 levels according to stereotactic section maps designed by Paxinos and Franklin^60^. Stereotaxic coordinates of the 12 regions of interest are expressed based on Bregma location as described previously^24^.

#### Measurement of infarct volume

Brain infarct volumes for the 12 stereotactic regions were determined by cresyl violet staining as previously described by our group^24^. The unstained area of brain sections was defined as infarcted. Infarct volumes (cortical, striatal, and total) were assessed by image analysis after digitization according to the method developed by Bordet et al.^61^ using an image analysis software (Saisam). Briefly, cortical and subcortical uncorrected infarcted areas and total hemispheric areas were calculated separately for each coronal slice. Total, cortical, and subcortical infarct volumes and hemispheric volumes (mm^3^) were calculated by the use of numerical integration of the respective areas for all the sections per animals and the distance between them. A corrected total infarct volume was calculated to compensate for the effect of brain edema. The corrected volume was calculated using the following equation: corrected infarct volume = infarct volume − (right hemisphere volume − left hemisphere volume).

#### Immunohistochemical examination of the ischemic area

Immunohistological analyses were performed on the brain region displaying the coordinates: Bregma 0.00 mm, as previously described^24^. Immunostainings were performed using rabbit polyclonal IgG anti-NeuN, (Abcam ab104225, 1:500), goat polyclonal IgG anti-Iba1 (Abcam ab5076, 1:500), and rabbit polyclonal IgG anti-GFAP (Abcam ab7260, 1:500) as detailed in Supplementary Methods. For each animal, quantification was performed using two different images of 90,000 µm^2^ (two images captured within the striatum and two within the cortex). Each image was gridded and three fields of 10,000 µm^2^ were randomly selected. The number of positive cells per field was counted in the cortex. Data are presented as the number of positive cells/field. Quantification was performed using Histolab software (version 6.0.5, Microvision Instruments-Evry).

### TUNEL assay

The In-Situ Cell Death detection kit (Roche, cat. No. 11684817910) was used to detect individual apoptotic cells in frozen brain sections. For each animal, staining was quantified based on a single image, captured using a 5 × objective (which represents a brain surface of 5 mm^2^, i.e. approximately one quarter of the total brain surface of the section), using Histolab software (version 6.0.5, Microvision Instruments-Evry). The total cortical and striatal surfaces, as well as the percent of TUNEL-positive surface in each zone, were measured in each image. A detailed protocol is presented in Supplementary Methods.

### Real-time PCR

Total RNA from ischemic (ipsilateral) hemispheres was isolated using mirVana PARIS Kit (Fisher Scientific, Illkirch, France) following the manufacturer’s instructions. As previously described by our group^24^, first strand cDNA was reverse transcribed from 1 µg total RNA with the High Capacity cDNA Reverse Transcription Kit (Applied Biosystems, Foster City, CA, USA). Real-time PCR reactions were performed in triplicate on a StepOnePlus real-time PCR system (Applied Biosystems) using 2 × SYBR green qPCR master mix (Applied Biosystems). The parameters for qPCR are presented in Supplementary Methods. The sequences of the PCR primers for each gene are presented in Supplementary Table 1. Levels of mRNA were normalized to that of the endogenous control, β-actin, and were calculated based on the fold change relative to the ipsilateral hemispheres of vehicle-treated SHAM mice.

### Western blotting

Total protein from ischemic (ipsilateral) hemispheres was isolated using the mirVana PARIS Kit (Fisher Scientific, Illkirch, France) following the manufacturer’s instructions. Western blotting was performed using rabbit polyclonal anti-phosphorylated AMPK α1/2 (1:500, Santa Cruz Biotechnology, Santa Cruz, CA, USA), rabbit monoclonal anti-phosphorylated ACC (ser-79) (1:1000, Cell Signaling Technology, Danvers, MA), rabbit monoclonal anti-ACC (1:1000, Cell Signaling Technology, Danvers, MA), rabbit monoclonal anti-phosphorylated NFκB p65 (1:1000, Cell Signaling Technology, Danvers, MA), rabbit polyclonal anti-IƘBα (1:1000, Cell Signaling Technology, Danvers, MA), or rabbit polyclonal anti-LC3A/B (1:1000, Cell Signaling Technology, Danvers, MA) as described previously^24^.

### Statistical analyses

Results are presented as box and whisker plots showing the median, interquartile, and variability outside the upper and lower quartiles. Data concerning the ischemic volumes and immunohistochemistry were obtained from a first cohort containing 7 SHAM-vehicle, 8 SHAM-metformin, 18 CKD-vehicle, and 14 CKD-metformin animals. Differences in the ischemic volumes and immunohistochemistry between groups were analyzed using a non-parametric Kruskall–Wallis test followed by Dunn’s multiple comparison post-hoc test. Neurobehavioral impairments, western blot and real-time PCR analyses were performed on samples collected from a second cohort of 14 SHAM-vehicle, 15 SHAM-metformin, 13 CKD-vehicle and 14 CKD-metformin animals. Results of the real-time PCR and western blots for each group are expressed as the ratio relative to the mean values measured in the ipsilateral hemisphere of the SHAM-operated mice. Differences in mRNA and protein levels between groups were analyzed using a non-parametric Mann Whitney test. Statistical analysis of neurobehavioral impairment was performed using a non-parametric Kruskall–Wallis test followed by Dunn’s multiple comparison post-hoc test. Data concerning hematology and serum calcium, phosphorus and metformin levels were obtained from the second cohort. Serum urea and lactate levels were measured in animals from both cohorts. Differences between groups were analyzed using a non-parametric Kruskall–Wallis test followed by Dunn’s multiple comparison post-hoc test. The threshold for statistical significance was set at p ≤ 0.05. All statistical analyses were performed using Graphpad Prism software (Graphpad). Correlations between variables were calculated using Spearman’s non-parametric correlation test. A schematic summary of the experimental protocol is presented in Supplementary Figure 1.

## Supplementary Information

Supplementary Information.
