# Supplementary material for: Metformin prevents stroke damage in non-diabetic female mice with chronic kidney disease
Source: Sci Rep. 2021 Apr 2;11:7464. doi: 10.1038/s41598-021-86905-9 (PMC8018962; doi:10.1038/s41598-021-86905-9)
Supplement: Supplementary file 1 — Supplementary Information. [file 41598_2021_86905_MOESM1_ESM.pdf]

Supplementary Information

Metformin prevents stroke damage in non-diabetic female mice with chronic kidney disease

Authors: Maria Grissi[1], Cédric Boudot[1], Maryam Assem[1,2,3], Alexandre Candellier[1,2,3], Mathilde Lando[1,2,3], Sabrina Poirot-Leclercq[1], Agnès Boullier[1,2,4], Youssef Bennis[1,5], Gaëlle Lenglet[1], Carine Avondo[1], Jean-Daniel Lalau[2,6,7], Gabriel Choukroun[1,2,3], Ziad A. Massy[8,9,10], Saïd Kamel[1,4,11], Jean-Marc Chillon[1,11,12] and Lucie Hénaut[1].

1. UR UPJV 7517 MP3CV, CURS, Amiens, 80054 France
2. Faculty of Medicine, University of Picardie Jules Verne, Amiens, 80000 France.
3. Division of Nephrology, Amiens University Hospital, 80054 France
4. Department of Biochemistry, Amiens University Hospital, 80054 France
5. Department of Clinical Pharmacology, Amiens University Hospital, 80054 France
6. Department of Endocrinology-Diabetology-Nutrition, Amiens University Hospital, 80054 France
7. UMR_I 01, PériTox, CURS, Amiens, 80054 France
8. Department of Nephrology, Ambroise Paré University Hospital, APHP, Boulogne-Billancourt, 92104 France
9. Inserm U1018 –Team 5, CESP, UVSQ, University Paris Saclay, Villejuif, 94807 France
10. University Versailles-Saint Quentin, University Paris-Saclay, Villejuif 91190, France
11. Faculty of Pharmacy, University of Picardie Jules Verne, Amiens, 80000 France
12. Direction of Clinical Research, Amiens University Hospital, 80054 France

Table of contents for the supplemental material

Supplementary Figure 1. Schematic illustration of the experimental protocol.

Supplementary Figure 2. Striatal apoptosis is not modified by metformin in mice with CKD.

Supplementary Figure 3. Metformin pre-conditioning has no impact on TNF-α expression in the ischemic lesions of CKD mice.

Supplementary Figure 4. Correlations between plasma urea levels, AMPK phosphorylation, NFKB activation, and macrophage/microglia M_1 marker levels.

Supplementary Figure 5. Real-time PCR analysis of ICAM-1, VCAM-1 and MCP1 mRNA expression.

Supplementary Figure 6. Metformin pre-conditioning enhances autophagy in ischemic lesions of SHAM but not CKD mice.

Supplementary Figure 7. Metformin pre-conditioning does not affect neurogenesis.

Supplementary Table 1. qRT-PCR primer sequences.

Raw western blot data associated with Figure 4

Raw western blot data associated with Supplementary Figure 6

Supplementary Methods

- Transient middle cerebral artery occlusion (tMCAO)
- Neurological evaluation
- Immunohistochemical examination of the ischemic area
- TUNEL assay
- Real-time PCR

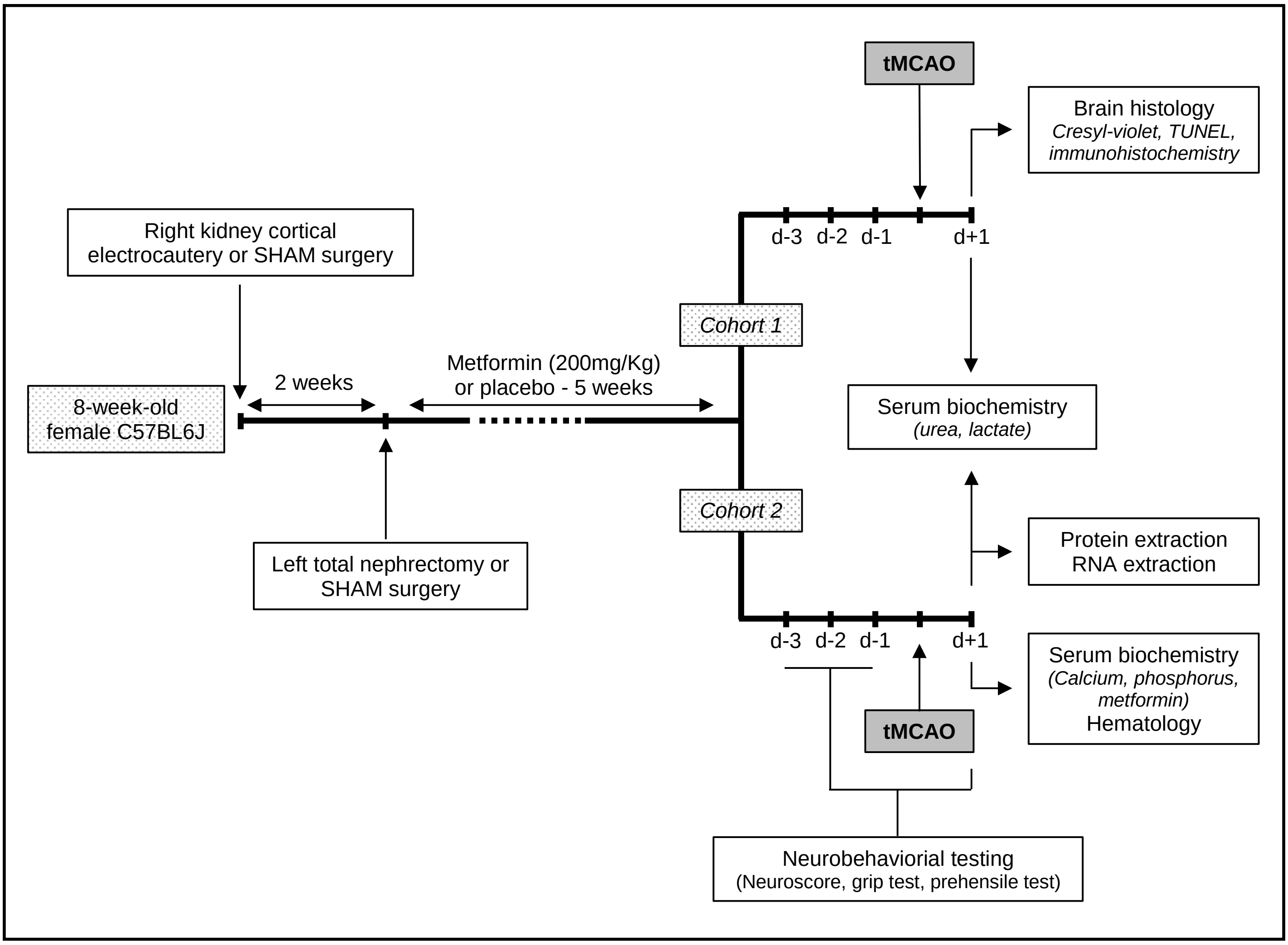

Supplementary Figure 1. Schematic illustration of the experimental protocol.

Abbreviations: tMCAO: transient middle cerebral artery occlusion.

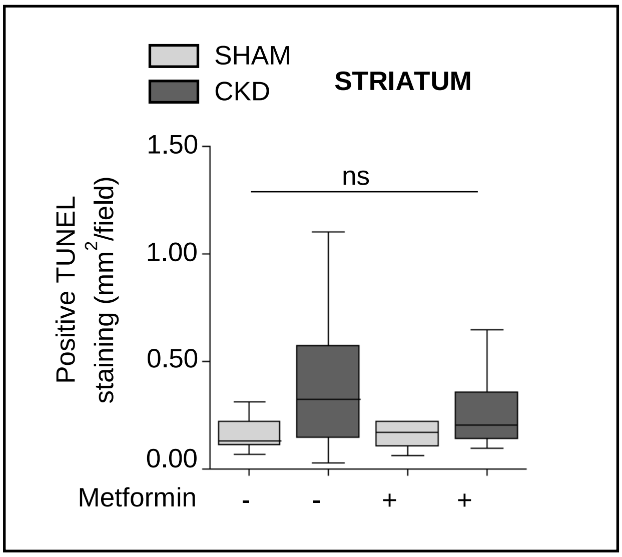

<u>Supplementary Figure 2</u>. Striatal apoptosis is not modified by metformin in mice with CKD. Quantification of TUNEL immunostaining of the striatum (TUNEL-positive surface per 40,000-µm^2 field). Results are expressed as the median, interquartile, and min-max and show data from at least 8 animals per group. Statistical analysis was performed using a non-parametric Kruskall-Wallis test followed by Dunn’s multiple comparison post-hoc test.

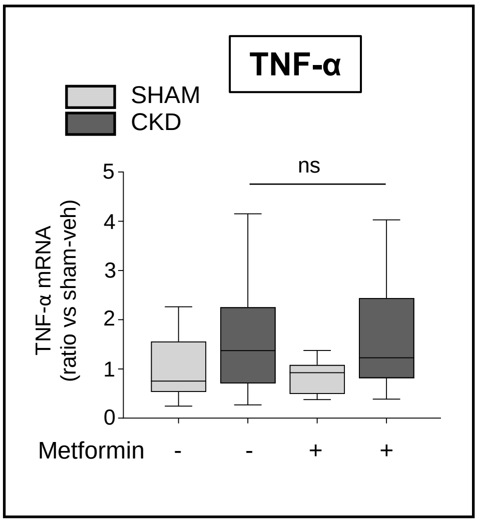

<u>Supplementary Figure 3.</u> Metformin pre-conditioning has no impact on TNF-α expression in the ischemic lesions of CKD mice. Results are expressed as the median, interquartile, and min-max and show data from at least 8 animals per group. Statistical analysis was performed using a non-parametric Kruskall-Wallis test followed by Dunn's multiple comparison post-hoc test.

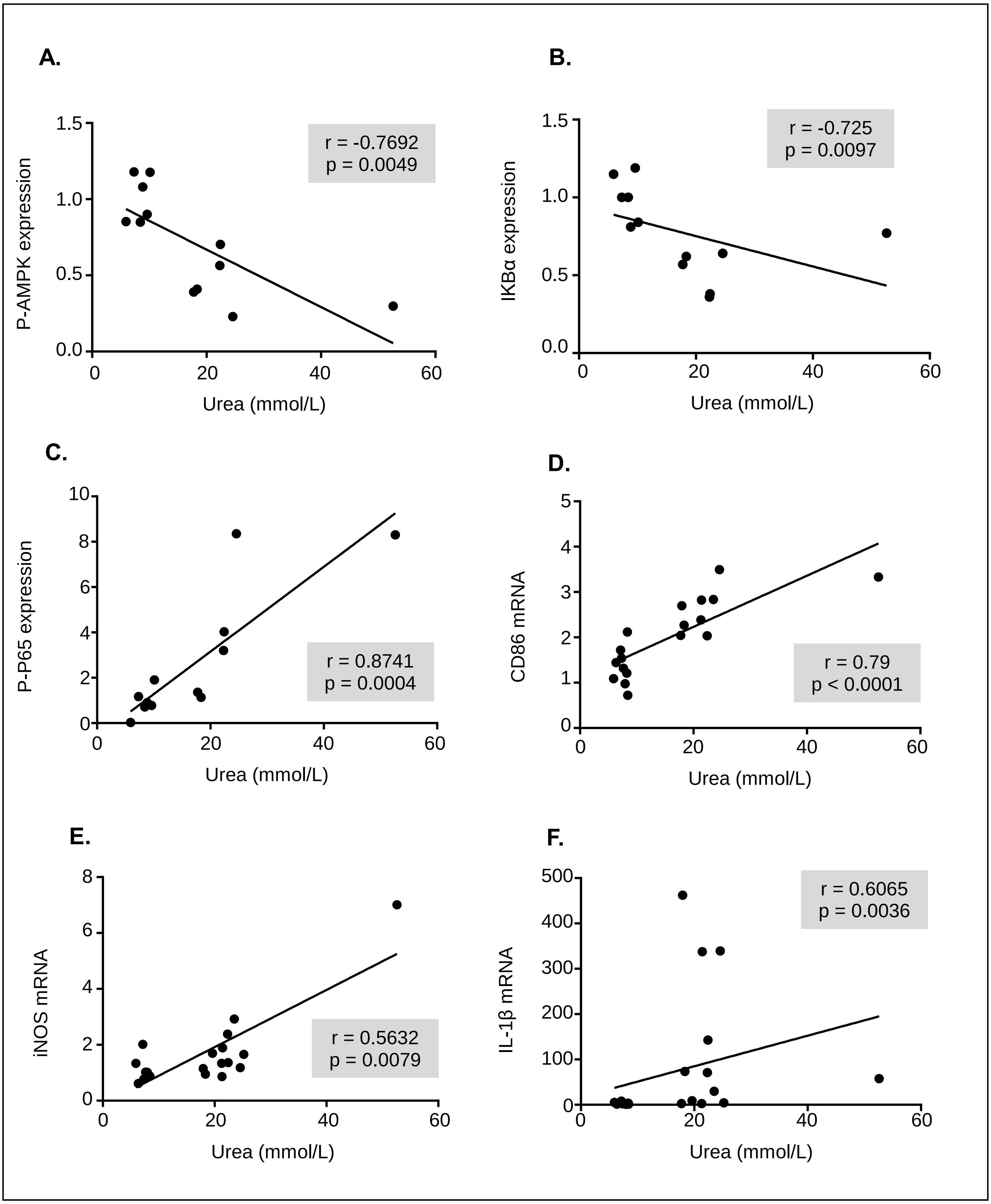

<u>Supplementary Figure 4.</u> Correlations between plasma urea levels, AMPK phosphorylation, NFKB activation, and macrophage/microglia M$_1$ marker levels. A. Correlation between plasma urea levels and the degree of AMPK phosphorylation (n = 6

SHAM-veh/n = 6 CKD-veh). **B.** Correlation between plasma urea levels and IKB degradation (n = 6 SHAM-veh/n=6 CKD-veh). **C.** Correlation between plasma urea levels and P65 phosphorylation (n = 6 SHAM-veh/n = 6 CKD-veh). **D.** Correlation between plasma urea levels and CD86 mRNA levels (n = 9 SHAM-veh/n = 9 CKD-veh). **E.** Correlation between plasma urea levels and iNOS mRNA levels (n = 9 SHAM-veh/n = 12 CKD-veh). **F.** Correlation between plasma urea levels and IL-1β mRNA levels (n = 9 SHAM-veh/n = 12 CKD-veh)**.** Statistical analysis was performed using a non-parametric Spearman correlation test.

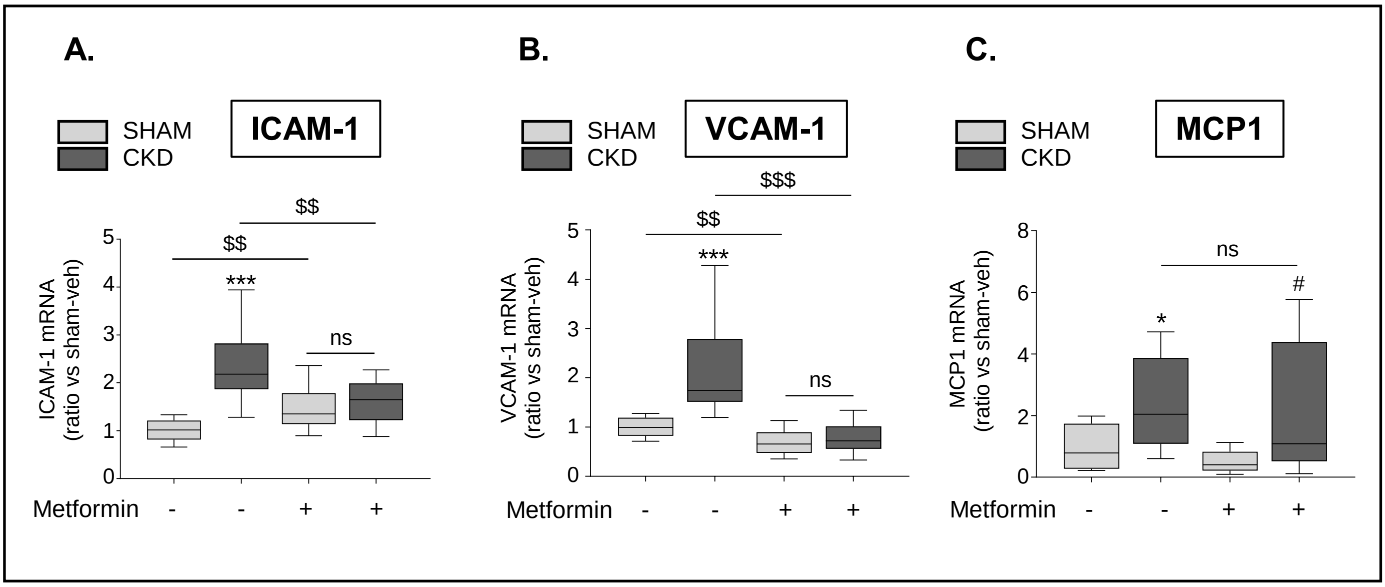

<u>Supplementary Figure 5.</u> Real-time PCR analysis of ICAM-1, VCAM-1 and MCP1 mRNA expression. Results are expressed as the median, interquartile, and min-max and show data from at least 8 animals per group. Statistical analysis was performed using a non-parametric Kruskall-Wallis test followed by Dunn's multiple comparison post-hoc test. $^{*}p < 0.05$, $^{***}p < 0.001$, CKD-vehicle *vs* SHAM-vehicle mice. $^{\#}p < 0.05$, CKD-metformin *vs* SHAM-metformin mice. $^{\$}p < 0.05$, $^{\$\$}p < 0.01$, $^{\$\$\$}p < 0.001$, CKD-metformin *vs* CKD-vehicle mice.

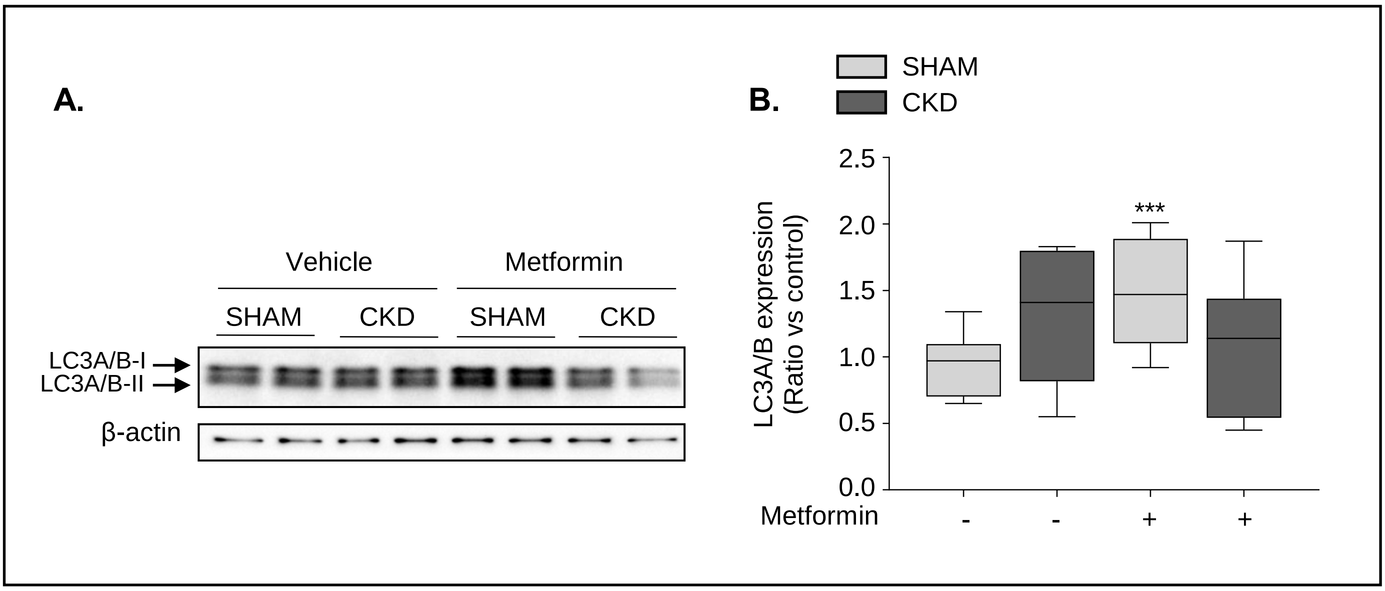

<u>Supplementary Figure 6.</u> **Metformin pre-conditioning enhances autophagy in ischemic lesions of SHAM but not CKD mice. A.** Representative images of LC3A/B-I and LC3A/B-II western blots performed on SHAM and CKD mice exposed, or not, to metformin. **B.** Quantitative data showing higher LC3A/B-I and LC3A/B-II expression in ischemic hemispheres of SHAM-metformin than SHAM-vehicle treated mice. No difference was observed between CKD mice exposed to metformin or the vehicle. Results are expressed as the median, interquartile, and min-max and show data from at least 6 animals per group. Statistical analysis was performed using a non-parametric Kruskall-Wallis test followed by Dunn's multiple comparison post-hoc test. ***p < 0.001, SHAM-metformin *vs* SHAM-vehicle mice.

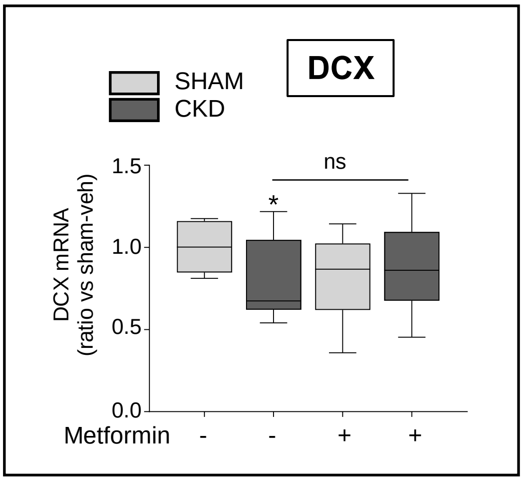

<u>Supplementary Figure 7.</u> Metformin pre-conditioning does not affect neurogenesis. Real-time quantitative PCR analysis of the mRNA expression of the immature neuronal marker DCX. Results are expressed as the median, interquartile, and min-max and show data from at least 8 animals per group. Statistical analysis was performed using a non-parametric Kruskall-Wallis test followed by Dunn's multiple comparison post-hoc test. *p < 0.05, CKD-vehicle *vs* SHAM-vehicle mice.

Gene	Forward	Reverse	Roles
**iNOS**	CCC TTC AAT GGT TGG TAC ATG G	ACA TTG ATC TCC GTG ACA GCC	Markers of classically activated (M1) macrophages/microglia
**TNFα**	ATG AGA AGT TCC CAA ATG GCC	CCA CTT GGT GGT TTG CTA CGA	
**IL-1**	ACC TTC CAG GAT GAG GAC ATG A	CTA ATG GGA ACG TCA CAC ACC A	
**IL-6**	GCT GGT GAC AAC CAC GGC CT	AGC CTC CGA CTT GTG AAG TGG T	
**CD32**	AAT CCT GCC GTT CCT ACT GAT C	GTG TCA CCG TGT CTT CCT TGA G	
**CD16**	TAC-ACA-GCA-CCA-GTC-CAA-GC	AGA-AAT-AAA-GGC-CCG-TGT-CC	
**CD86**	GAG CGG GAT AGT AAC GCT GA	GGC TCT CAC TGC CTT CAC TC	
**Fizz1**	ATG ACT GCT ACT GGG TGT GC	GCA GTG GTC CAG TCA ACG AG	Markers of alternatively activated (M2) macrophages/microglia
**CD206**	TCT-TTG-CCT-TTC-CCA-GTC-TCC	TGA-CAC-CCA-GCG-GAA-TTT-C	
**ARG I**	GAA CAC GGC AGT GGC TTT AAC	TGC TTA GCT CTG TCT GCT TTG G	
**ICAM-1**	CCT GTT TCC TGC CTC TGA AG	GTC TGC TGA GAC CCC TCT TG	Markers used to study monocytes infiltration and blood-brain barrier disruption
**VCAM-1**	CCC AAG GAT CCA GAG ATT CA	TAA GGT GAG GGT GGC ATT TC	
**MCP1**	ATG CAG TTA ACG CCC CAC T	CAT TCC TTC TTG GGG TCA GC	
**DCX**	AGC TGA CTC AGG TAA CGA CCA	GCT TTG ACT TAG GTG TTG AGA GC	Neurogenesis marker

Supplementary Table 1. qRT-PCR primer sequences.

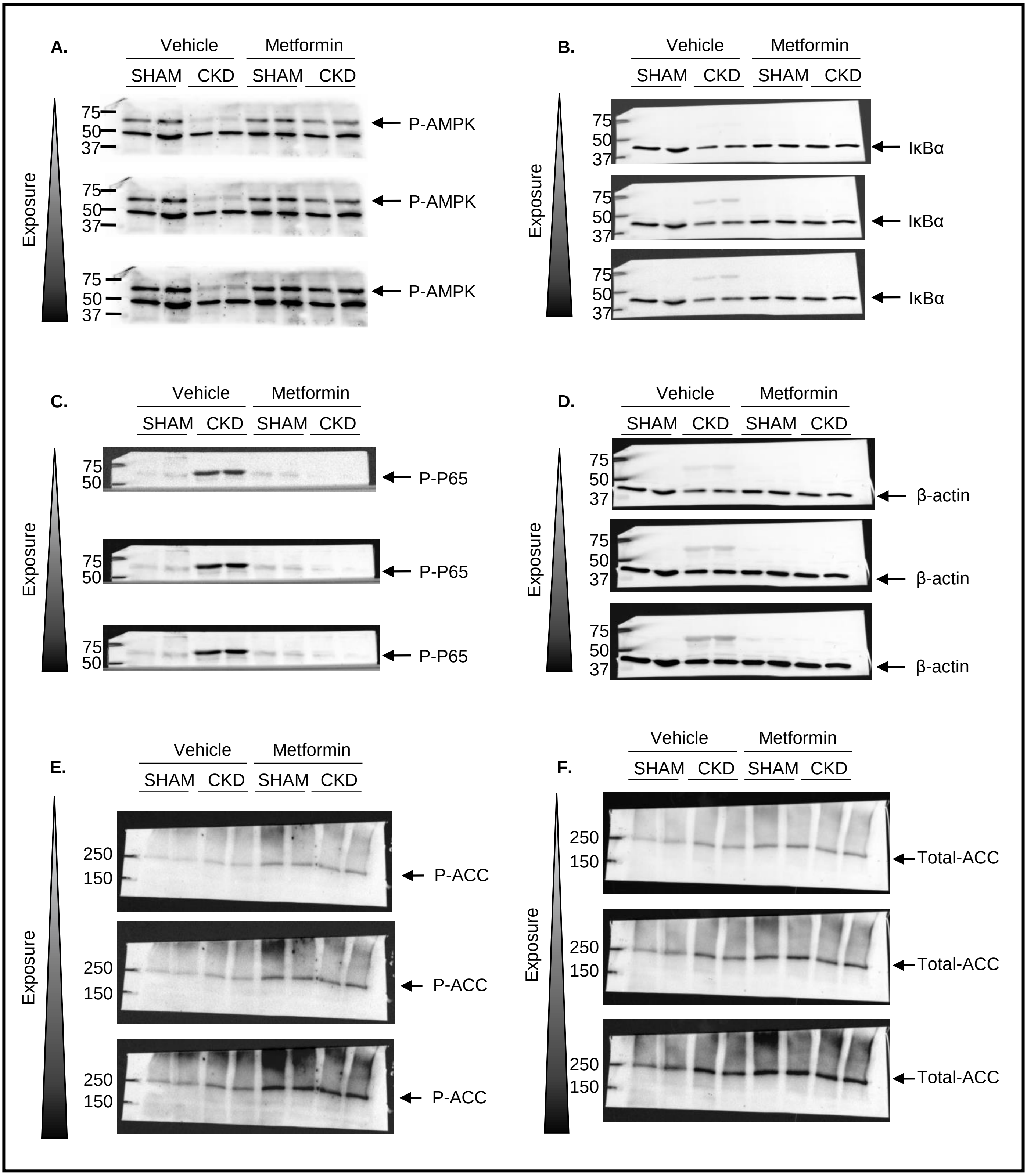

Raw western blot data associated to Figure 4: A. B. C. D. E. and F. Multiple exposures of full-length blots showing AMPK phosphorylation, IκBα degradation, P65 phosphorylatio, β-actin expression, ACC phosphorylation and total-ACC expression respectively.

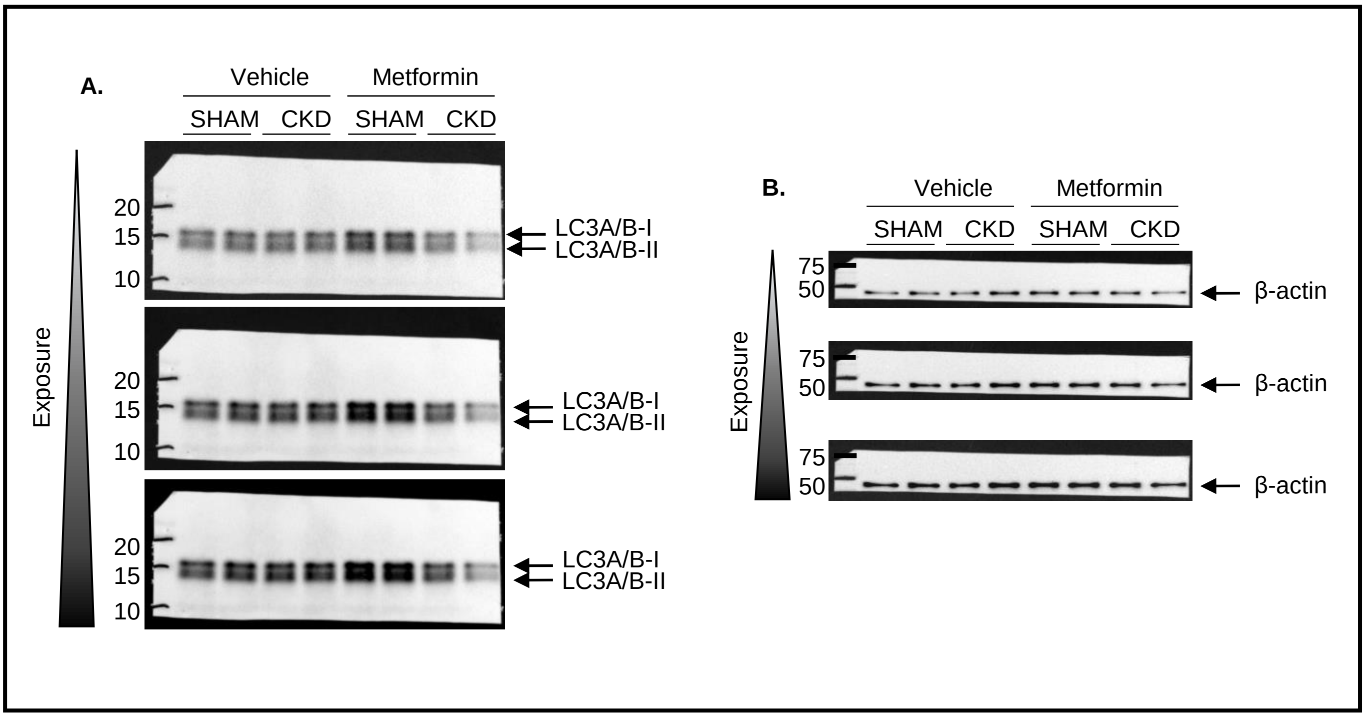

Raw western blot data associated to Supplementary Figure 6: A and **B.** Multiple exposures of full-length blots showing LC3A/B-I and LC3A/B-II (A.) and β-actin expression (B.).

Supplementary Methods

Transient middle cerebral artery occlusion (tMCAO)

Ischemic lesions were induced in both SHAM and CKD mice 5 weeks after the last SHAM or CKD surgery (*i.e.* in 15-week-old mice). Briefly, mice were anesthetized with ketamine (80 mg/kg) plus xylazine (8 mg/kg) and a 20-mm-long 6-0 silicon rubber–coated nylon monofilament (Doccol®, Sharon, Massachusetts, USA) was inserted into the right common carotid artery. The diameter of the coated filaments was 0.21 ± 0.02 mm (reference 6021910PK5Re). MCAO sutures in this category of coated tip diameter are suitable for MCAO in animals with a body weight in the range of 22 ± 2 g. The filament was then advanced to the internal carotid artery and passed into the intracranial circulation (12–13 mm distal to the carotid bifurcation), thus occluding the origin of the MCA. The right MCA was occluded for 15 min. The filament was then carefully removed to produce the reperfusion. Animals were allowed to recover for 24 h and then euthanized for infarct volume analysis.

Neurological evaluation

- Neuroscore: A six-grade neuroscore was used to assess post-ischemic motor and behavioral impairments. Mice were graded 0 to 5 as follows. **Grade 5**: mice were held gently by the tail one meter above the ground and observed for forelimb flexion. Normal mice extended both forelimbs toward the floor. Mice that extended both forelimbs toward the floor and did not display other neurological impairment were assigned a grade of 5. **Grade 4**: mice with consistent flexion of the forelimb contralateral to the injured hemisphere (varying from mild wrist flexion and shoulder adduction to severe posturing, with full flexion of the wrist and elbow, and induction of the shoulder with internal rotation) were assigned a grade of 4. **Grade 3:** mice were placed on a large sheet of soft, plastic-coated paper that they could grip firmly

with their claws. The experimenter held the mouse by the tail and applied gentle lateral pressure to the animal's shoulder until the forelimbs slid several centimeters. The maneuver was repeated several times to the left and to the right. Normal mice and slightly impaired mice resisted sliding to an equivalent extent in each direction. However, severely impaired mice with consistently reduced resistance to pushing towards the paretic side were assigned a grade of 3. **Grade 2**: mice were then allowed to move about freely and observed for circling behavior when their tail was pulled. Mice that circled consistently towards the paretic side were assigned a grade of 2. **Grade 1**: mice were allowed to move about freely and were observed for circling behavior. Mice that circled spontaneously and consistently toward the paretic side were assigned a grade of 1. **Grade 0**: mice without any spontaneous motion were assigned a grade of 0.

- Prehensile test: A prehensile test was performed using a horizontal stainless-steel wire (length: 60 cm, diameter: 3 mm) placed 40 cm above a foam pad. The wire was graded into 6 equal parts starting from the point of suspension till the platform on each side. The forepaws of the mice were placed on the wire in the centre and the animals were left suspended for 20 seconds. The time until the mice fell, their ability to grab the wire with a hind paw, and their motor coordination were measured. The tested animals were scored as follows: 1, 2, 3 or 4 points for holding onto the wire for less than 5 seconds, 6 to10 seconds, 11 to 15 seconds or more than 15 seconds respectively. Mice were scored 0 if they fell immediately after being suspended. An additional point was added if the mice managed to grab the wire with a hind paw. If they managed to advance to either side, mice were given 1, 2, 3, 4 or 5 points for reaching grading 1, 2, 3, 4 and 5 respectively. An extra point was granted if they were able to reach the platform.

- Grip-test: The muscular strength of the forelimbs was assessed using a grip strength test (Bioseb, Vitrolles, France). A grip strength meter was positioned horizontally and the mice were held by the tail and lowered towards the apparatus. The animals were allowed to grab the

metal grid and were then pulled backwards in the horizontal plane. The force applied to the grid just before the animals lost grip was recorded as the peak tension. The muscular strength of the forelimbs was assessed three times per session and the mean of the three measurements was used for evaluation.

Immunohistochemical examination of the ischemic area

The sections used for immunohistology were cut to a thickness of 20 µm, *i.e.* thinner than sections used for cresyl violet staining, to facilitate antibody penetration into the brain tissue. Sections were fixed with 4% ice-cold PFA for 5 min at room temperature (RT) and incubated in sodium citrate (1M, pH = 6) for 20 min at 100°C for antigen retrieval. Sections were then quenched in 100 mmol/L glycine in PBS for an additional 10 min and permeabilized for 1 h at RT with 0.3% triton X-100 in PBS containing 1% Bovine Serum Albumin (BSA). Non-specific binding of the antibody was blocked by incubation in a blocking solution (1% BSA in PBS) for 30 min at RT. Sections were then incubated overnight at 4°C with primary antibody (rabbit polyclonal IgG anti-NeuN, Abcam ab104225, diluted 1:500; goat polyclonal IgG anti-Iba1, Abcam ab5076, diluted 1:500; and rabbit polyclonal IgG anti-GFAP, Abcam ab7260, diluted 1:500) prepared in PBS containing 0.3% triton X-100 and 1% BSA. Brain sections were then rinsed and incubated with secondary antibody (Alexafluor® 488 goat anti-rabbit IgG, Invitrogen A11008; Alexafluor® 488 goat anti-rat IgG, Invitrogen A11004; Alexafluor® 488 goat anti-goat IgG, Invitrogen A11055; diluted 1:500), prepared in 1% BSA PBS, for 1 h at RT. Samples were then extensively washed in PBS. Nuclei were counterstained with Hoechst and samples were mounted with Mowiol solution (Mowiol® 4-88, Sigma-Aldrich, St. Quentin Fallavier, France) for fluorescence detection.

TUNEL assay

Briefly, 20-µm sections were fixed with 4% ice-cold PFA for 5 min at RT and incubated in sodium citrate (1M, pH = 6) for 20 min at 100°C for antigen retrieval. Sections were then quenched in 100 mmol/L glycine in PBS for an additional 10 min and permeabilized for 1 h at RT with 0.3% triton X-100 in PBS containing 1% BSA. Non-specific binding of the antibody was blocked by incubation in a blocking solution (1% BSA in PBS) for 30 min at RT. Samples were then incubated for 60 min at 37°C in the TUNEL reaction mixture (prepared according to the manufacturer's instructions) in a humidified atmosphere in the dark. Nuclei were counterstained with Hoechst and samples were mounted with Mowiol solution (Mowiol® 4-88, Sigma-Aldrich, St. Quentin Fallavier, France) for fluorescence detection.

Real-time PCR

Pre-amplification was performed with the following touchdown PCR protocol: 95°C for 10 min, 95°C for 15 s, 66°C for 1 min, 95°C for 15 s, 64°C for 1 min, 95°C for 15 s, 62°C for 1 min. This was followed by 40 cycles of 95°C for 15 s and 60°C for 1 min. The primer sequences used are listed in Supplementary Table 1.
